# Supplementary material for: Pharmacological Inhibition of Membrane Signaling Mechanisms Reduces the Invasiveness of U87-MG and U251-MG Glioblastoma Cells In Vitro
Source: Cancers (Basel). 2023 Feb 6;15(4):1027. doi: 10.3390/cancers15041027 (PMC9954756; doi:10.3390/cancers15041027)
Supplement: Supplementary file 1 [file cancers-15-01027-s001.zip › cancers-2185164-supplementary.pdf]

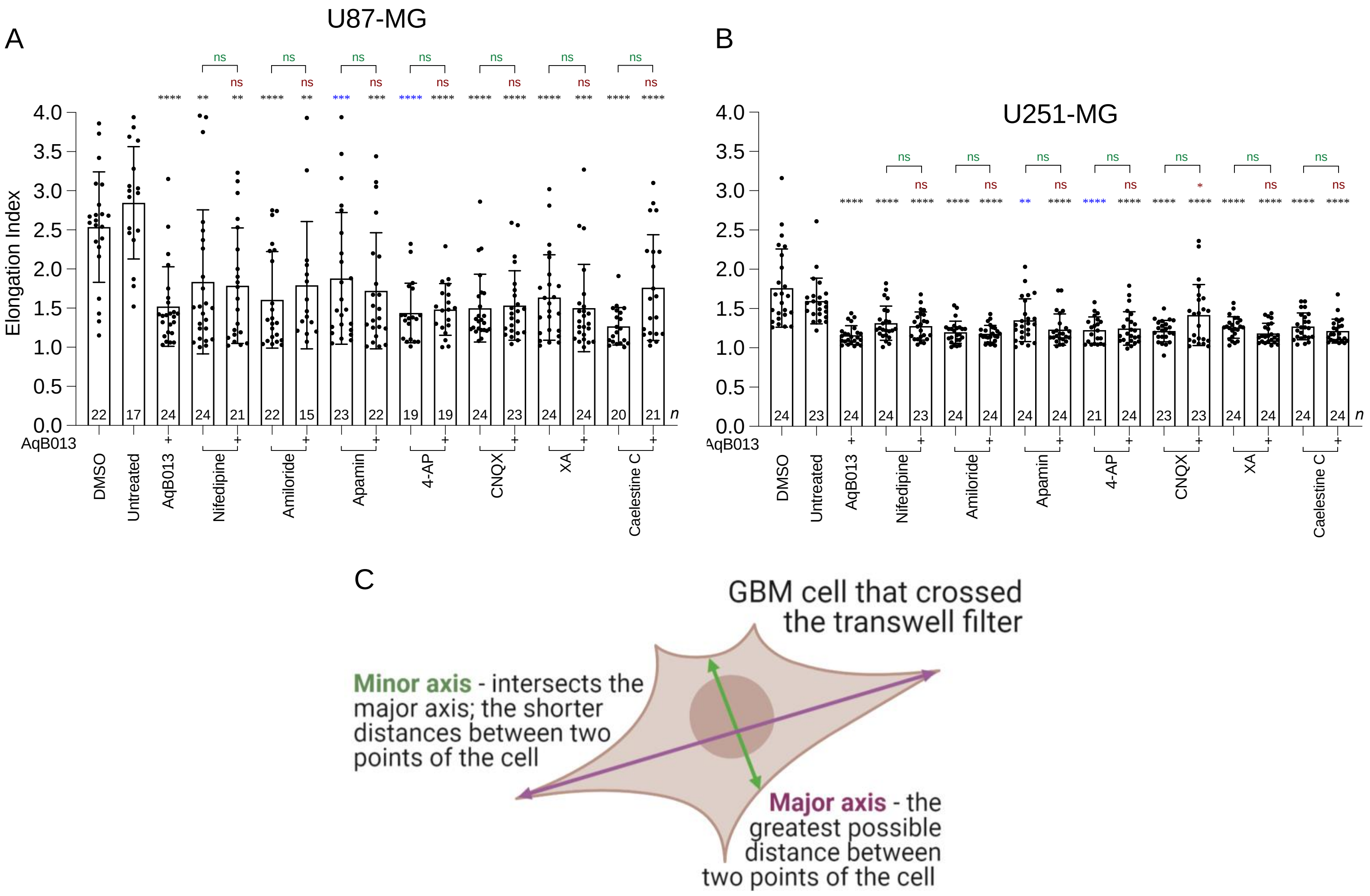


**Figure S1.** Effects of pharmacological channel inhibitors and natural compounds on morphology of U87-MG **(A)** and U251-MG (**B**) cells. Compiled data shown in bar histograms represent the elongation indexes of a subset of cells following treatment with drugs or controls (U87-MG, 4 hrs; U251-MG, 4.5 hrs). Elongation index was calculated as the ratio of the minor axis to the major axis of a minimum of 5 cells that had traversed the transwell filter across 3 fields of view for each treatment and control group. Statistically significant differences in elongation index between treatment groups were determined using one-way ANOVA with Dunnett's post-hoc tests; comparisons between treatments and matched controls are shown for vehicle (black) and untreated (blue). Differences for compounds with and without AqB013 are shown in green. Differences between combined treatments and AqB013 alone are in red. Doses of compounds were as specified in Figure 2. **(C)** Schematic diagram indicating the major and minor axes of invaded GBM cells that were measured to calculate the elongation indexes plotted in the histograms.

**Figure S2:** U87-MG spheroid perimeters measured at 0 and 96 h during treatments with DMSO (0.2%), AqB013 (28 µM), nifedipine (50 µM), amiloride (20 µM), apamin (20 µM), 4-AP (500 µM), CNQX (60 µM), XA (0.6 µM) or the same doses of each drug and AqB013 (28 µM) in combination. No reductions in spheroid growth rates (perimeter lengths, shown as grey outline) were seen.

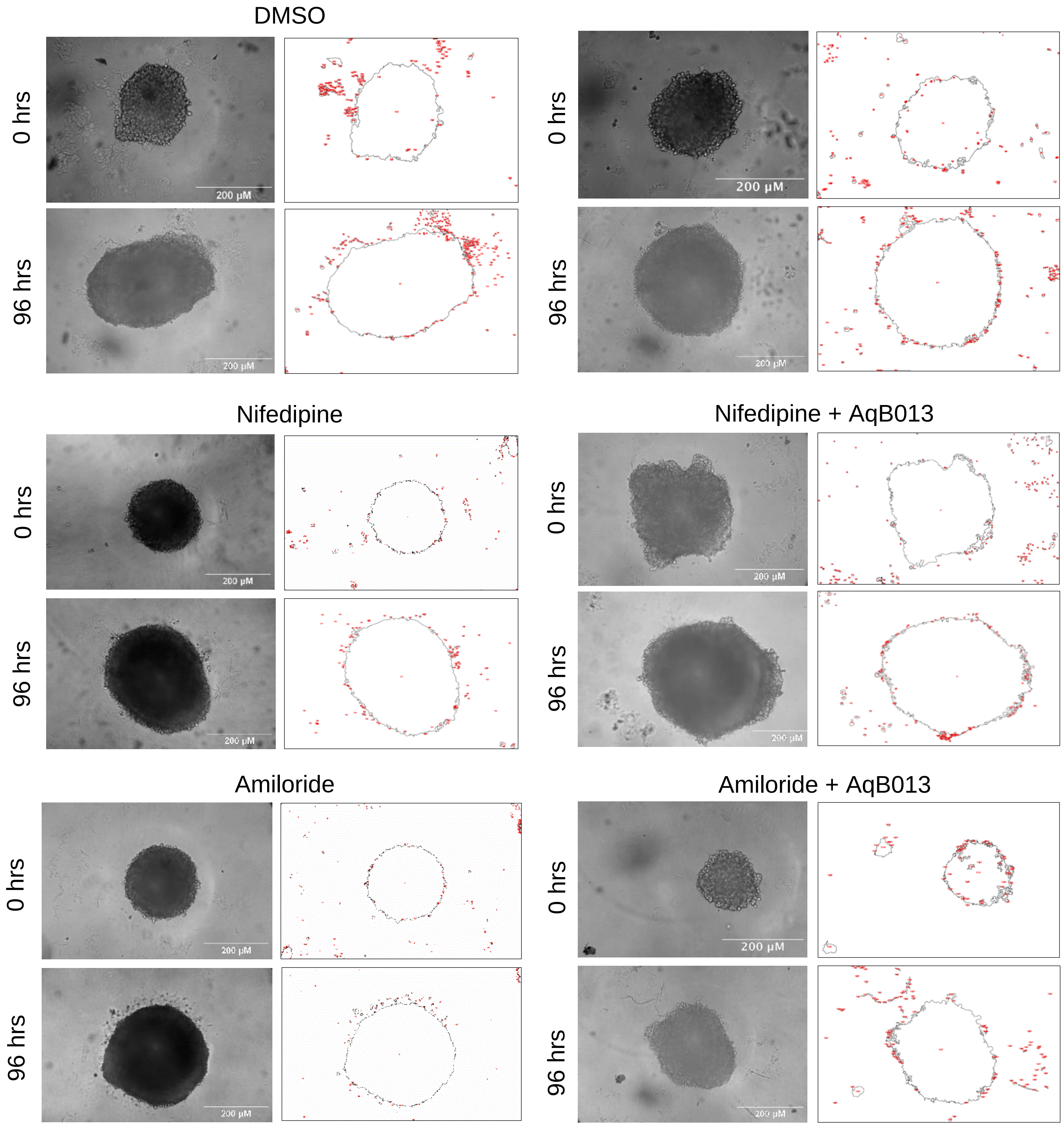

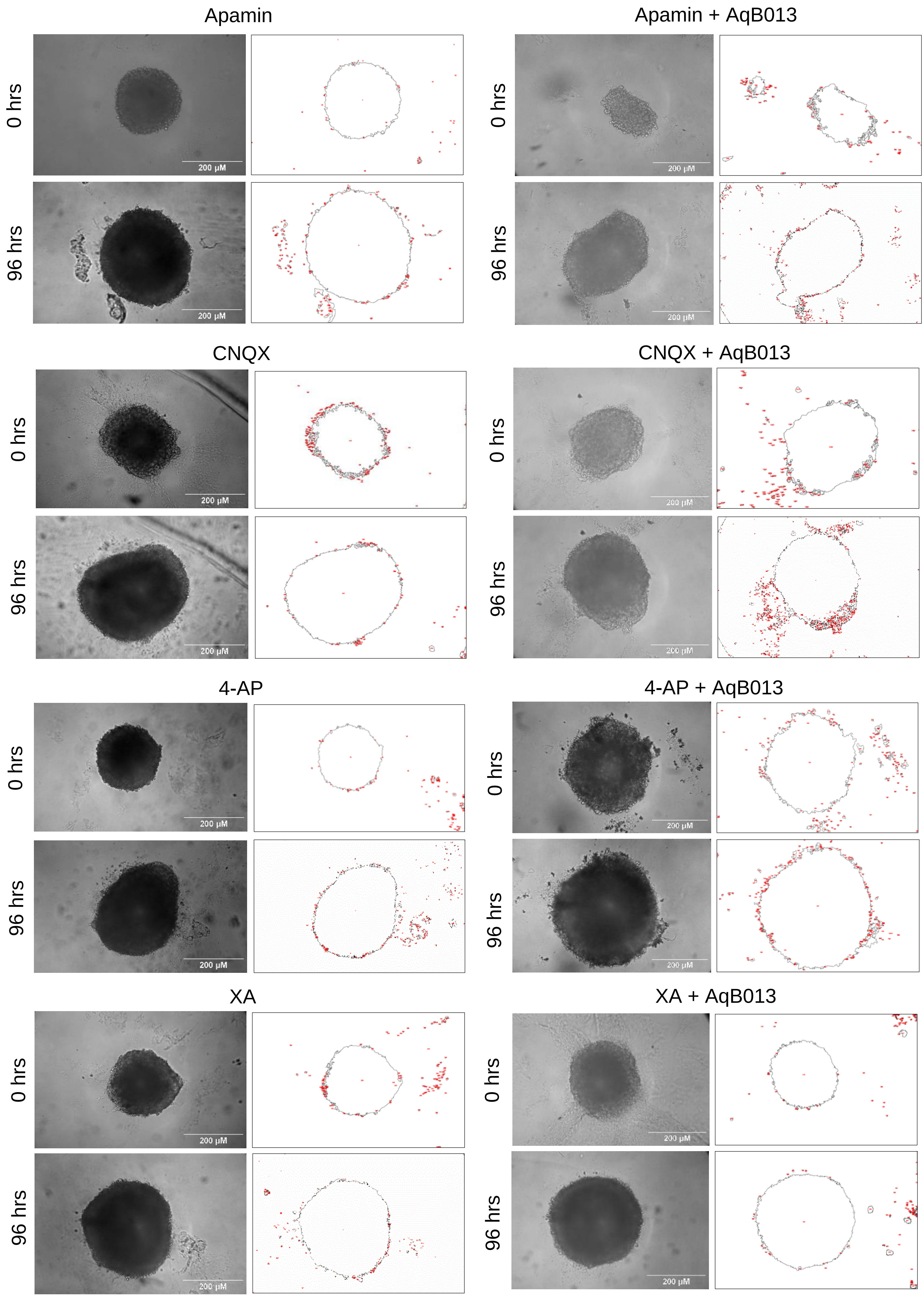

Apamin
Apamin + AqB013
CNQX
CNQX + AqB013
4-AP
4-AP + AqB013
XA
XA + AqB013
0 hrs
96 hrs
200 μM

**Figure S3:** U251-MG spheroid perimeters measured at 0 and 96 h during treatments with DMSO (0.2%), AqB013 (28 µM), nifedipine (50 µM), amiloride (20 µM), apamin (20 µM), 4-AP (500 µM), CNQX (60 µM), XA (0.6 µM) or the same doses of each drug and AqB013 (28 µM) in combination. No reductions in spheroid growth rates (perimeter lengths, shown as grey outline) were seen.

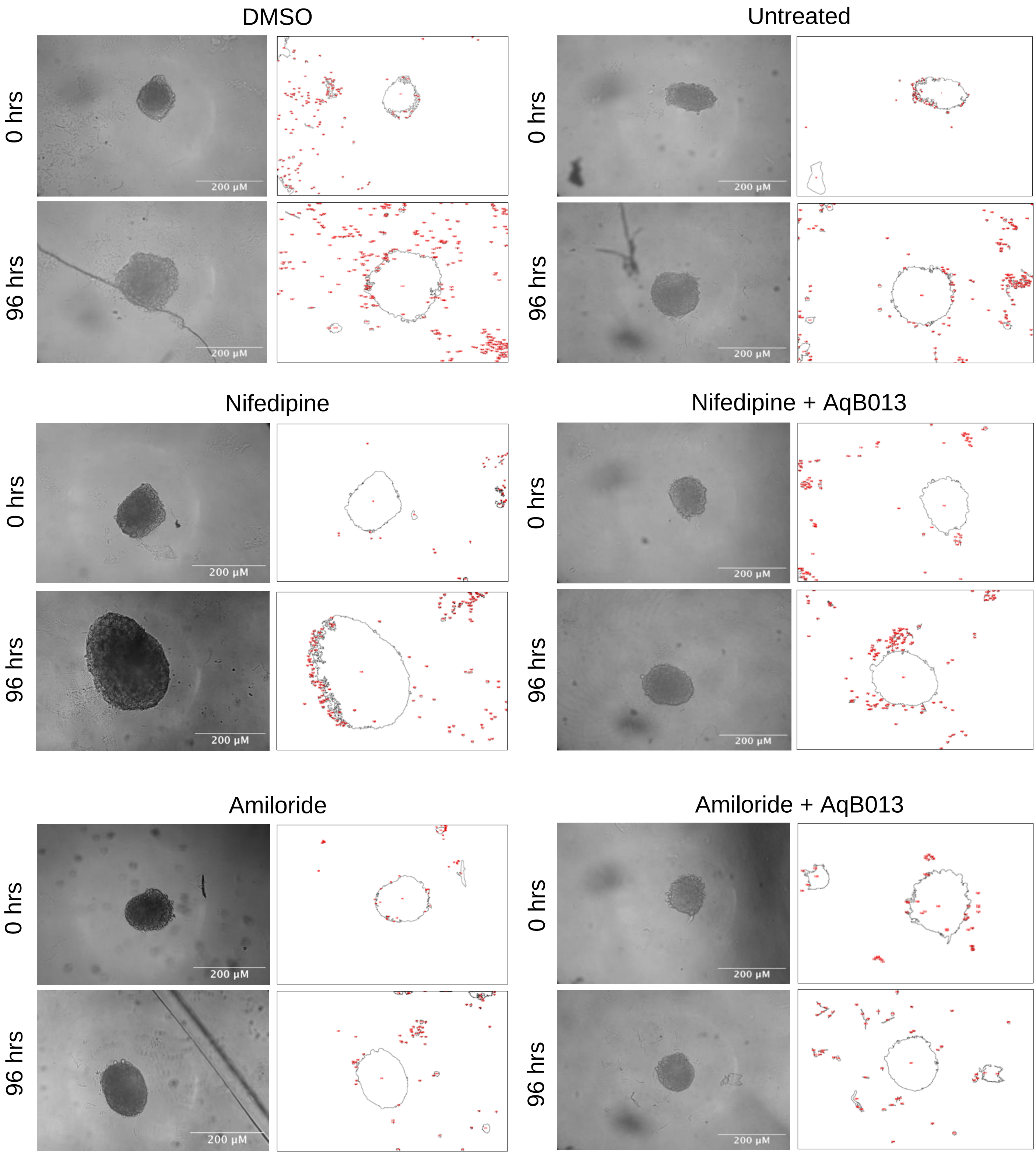

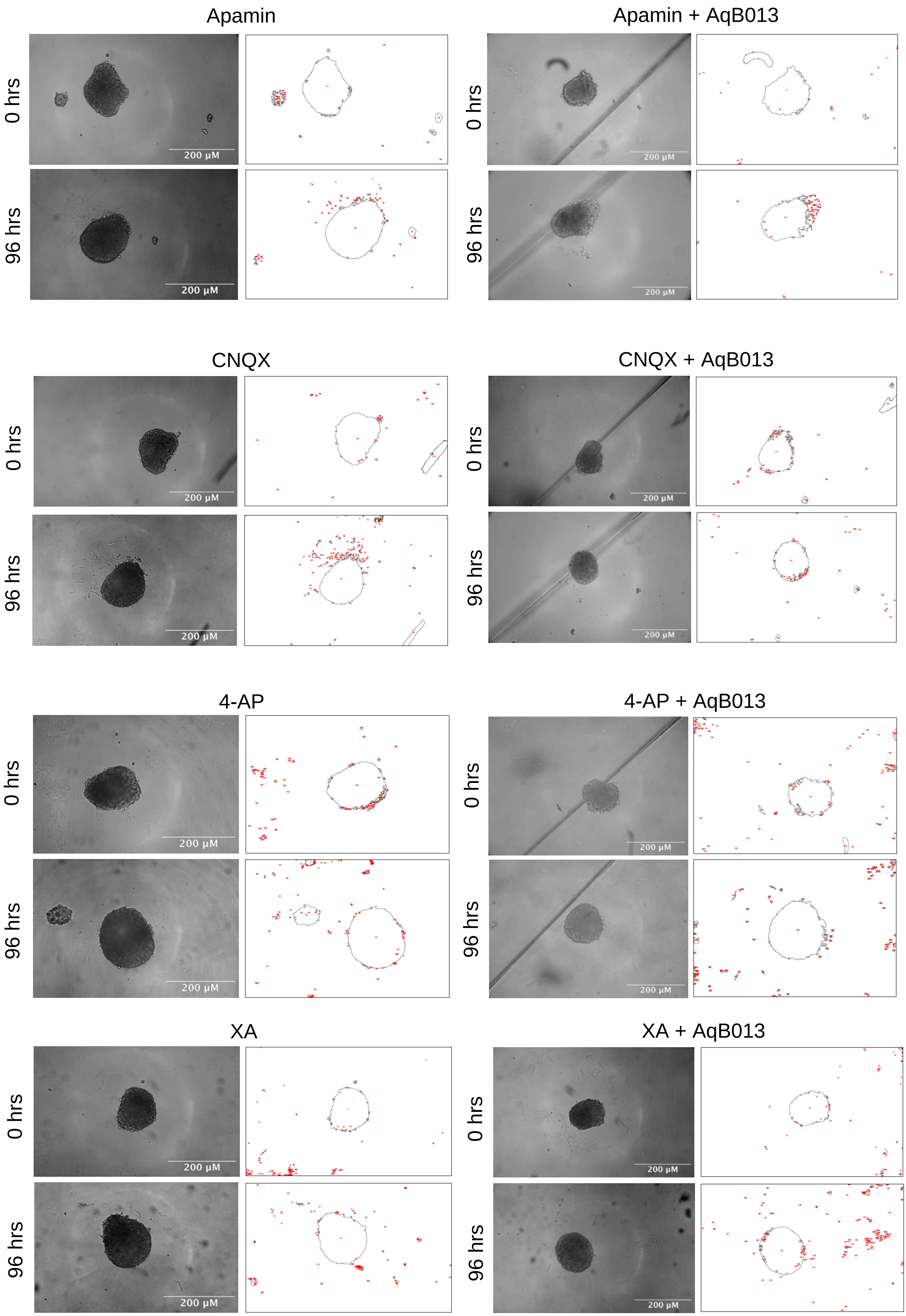

Apamin
Apamin + AqB013
CNQX
CNQX + AqB013
4-AP
4-AP + AqB013
XA
XA + AqB013
0 hrs
96 hrs
200 µM
